# Molecular Systems Architecture of Interactome in the Acute Myeloid Leukemia Microenvironment

**DOI:** 10.3390/cancers14030756

**Published:** 2022-02-01

**Authors:** V. A. Shiva Ayyadurai, Prabhakar Deonikar, Kevin G. McLure, Kathleen M. Sakamoto

**Affiliations:** 1 Systems Biology Group, International Center for Integrative Systems, Cambridge, MA 02138, USA; prabhakar@integrativesystems.org; 2 Ermaris Bio, Inc., Oakland, CA 94618, USA; kevin@ermarisbio.com; 3 Division of Hematology/Oncology, Department of Pediatrics, Stanford University, Stanford, CA 94305, USA; kmsakamo@stanford.edu

**Keywords:** acute myeloid leukemia (AML), leukemia stem cells (LSC), CytoSolve, systems biology, tumor microenvironment (TME), molecular systems architecture, immune cells

## Abstract

**Simple Summary:**

Acute myeloid leukemia (AML) is a cancer of blood and bone marrow that causes rapid production of abnormal red and white blood cells. Once established, the cancer cells communicate through a complex set of molecular interactions with neighboring cells in order to survive, spread rapidly, and evade detection and destruction by the body’s immune system. In this study, a systematic review produced a comprehensive set of critical molecular interactions that was then organized into molecular “systems architecture” to map the communications between cancer cells and neighboring cells. This systems architecture may aid in identifying effective targets that disrupt communication between the cancer cells and the neighboring environment, leading to effective treatment strategies.

**Abstract:**

A molecular systems architecture is presented for acute myeloid leukemia (AML) to provide a framework for organizing the complexity of biomolecular interactions. AML is a multifactorial disease resulting from impaired differentiation and increased proliferation of hematopoietic precursor cells involving genetic mutations, signaling pathways related to the cancer cell genetics, and molecular interactions between the cancer cell and the tumor microenvironment, including endothelial cells, fibroblasts, myeloid-derived suppressor cells, bone marrow stromal cells, and immune cells (e.g., T-regs, T-helper 1 cells, T-helper 17 cells, T-effector cells, natural killer cells, and dendritic cells). This molecular systems architecture provides a layered understanding of intra- and inter-cellular interactions in the AML cancer cell and the cells in the stromal microenvironment. The molecular systems architecture may be utilized for target identification and the discovery of single and combination therapeutics and strategies to treat AML.

## 1. Introduction

Acute Myeloid Leukemia (AML) is characterized by uncontrolled proliferation, increased survival, and impaired differentiation of hematopoietic progenitor cells [1]. Increased proliferation and apoptosis resistance, as well as the inhibition of differentiation and/or aberrant activation of growth factor receptor signaling pathways, are central to AML pathogenesis [2,3]. Aberrant and constitutive activation of signal transduction molecules are found in about 50% of primary AML bone marrow samples, enhancing the survival and proliferation of hematopoietic progenitor cells via the RAF/MEK/ERK cascade and the PI3K/AKT pathways that are dysregulated by mutations in receptor tyrosine kinases (RTK), Fms related receptor tyrosine kinase 3 (FLT3), N-Ras and K-Ras, and Kit [1,4].

Specific surface biomarkers characterize the subpopulations of AML cells. For example, leukemic stem cells are characterized by CD34^+^/CD38^−^ surface markers, megakaryocyte-erythroid progenitors (MEPs) are characterized by CD34^+^/CD38^+^/CD45RA^−^ surface markers, and granulocytic-monocytic progenitors (GMPs) are characterized by CD34^+^/CD38^+^/CD45RA^+^ surface markers [5,6,7]. Aberrant multipotent progenitor cells give rise to myeloid lineage-committed cells showing further phenotypical as well as functional changes. AML patients present with a significantly expanded population of granulocytic-monocytic progenitor cells (GMP), while the megakaryocytic-erythroid progenitor (MEP) population is severely reduced [8]. In addition, there is a significant depletion of HSC numbers in AML as a result of a differentiation block at the HSC–progenitor transition [9]. The increased constitutive activation of GMP clusters in AML has been attributed to insufficient production of cytokines such as TGFβ 1 and CXCL4—factors that promote the quiescence of the GMP clusters [10].

Gene mutations precipitate key events in AML pathogenesis [11,12]. The gene mutations common in AML are well documented elsewhere [7] and some of the key genetic factors are summarized in Table 1. Class I mutations lead to uncontrolled cellular proliferation and evasion of apoptosis and include mutation conferring constitutive activity to tyrosine kinases or dysregulation of downstream signaling molecules (in genes such as BCR-ABL, LLT3, c-KIT, and RAS) [13]. Class II mutations are associated with inhibition of differentiation, including key transcription factors, such as CBF and retinoic acid receptor alpha (RARα), and proteins that are involved in transcriptional regulation, such as p300, CBP, MOX, TIF2, and MLL [14]. Class III mutations are involved in epigenetic regulation and include genes such as TET2, IDH1/2, DNMT3A, ASXK1, Cohesin, NPM1 and EZH2 [11,14]. Additionally, genes such as WT1 and TP53 are implicated in tumor suppression activity [14]. Oncogenes common in AML include PML-RARa, FLT3-ITD, AML-ETO, and CBFB-MYH11 (oncogene for Inversion 16 cytogenetic alteration) [14,15].

Recent reviews have discussed in detail the cytogenetic targets for potential treatments of AML [14,16,17]. In addition to cytogenetic factors, the interactions in the tumor microenvironment that promote suppression of immune response, cancer cell proliferation, and inhibition of apoptosis also contribute significantly to the pathogenesis of AML [2]. This research employs a systems biology approach to provide not only a systematic review of the current understanding of AML tumor microenvironment (TME) but also a molecular systems representation, i.e., the interactome of the molecular interactions within cancer cells and across the cells in the stromal microenvironment. The insights from this review aim to provide the AML research community an integrative molecular systems approach to understanding the complexity of the biomolecular interactions involved in AML pathogenesis. The results of this investigation may be used to support identification of potential targets for therapeutic interventions.

## 2. Literature Review

The scientific literature was searched to identify journal papers that contain research on AML, molecular pathways of AML, cells in the AML microenvironment, interactions between AML cells and the cells of AML microenvironment, and the molecular pathways involved in the cellular crosstalk in the AML microenvironment. CytoSolve® is an established systems biology tool that enables a systematic bioinformatics literature review process, as well as providing scalable computational modeling of molecular pathways [18,19,20,21,22,23,24]. In this study, CytoSolve has been employed to perform a systematic review as well as to support the curation and development of the molecular systems architecture of AML pathogenesis.

The systematic review process for this study involved the following four steps:

1. creating a list of Medical Subject Headings (MeSH) keywords to optimize the recall and precision of peer-reviewed articles (listed in Table 2);
2. searching and retrieving the relevant peer-reviewed articles published between January 1980 to June 2021 from PubMed, Medline, and Google Scholar, which were stored as an “Initial Set” repository;
3. screening the titles and abstracts of the articles in the Initial Set repository to identify the most relevant articles based on our inclusion criteria, which were stored as the “Final Set” repository; and
4. performing a full-length review of the Final Set repository using the domain experts.

### The Inclusion Criteria

The full text of the articles, not only the abstracts, were reviewed completely by the authors. An article was deemed relevant only if the body of the article contained the keywords set out in Table 2 (e.g., CXCR4, TGF-β, MDSC, etc.), with specific relation to AML pathogenesis. In the screening process, abstracts and unpublished literature were not sought, as they had not been peer-reviewed adequately to authenticate their results. The List of Medical Subject Headings (MeSH) keywords to optimize recall and precision of peer-reviewed articles is provided in Table 2 below.

**Figure 1:**
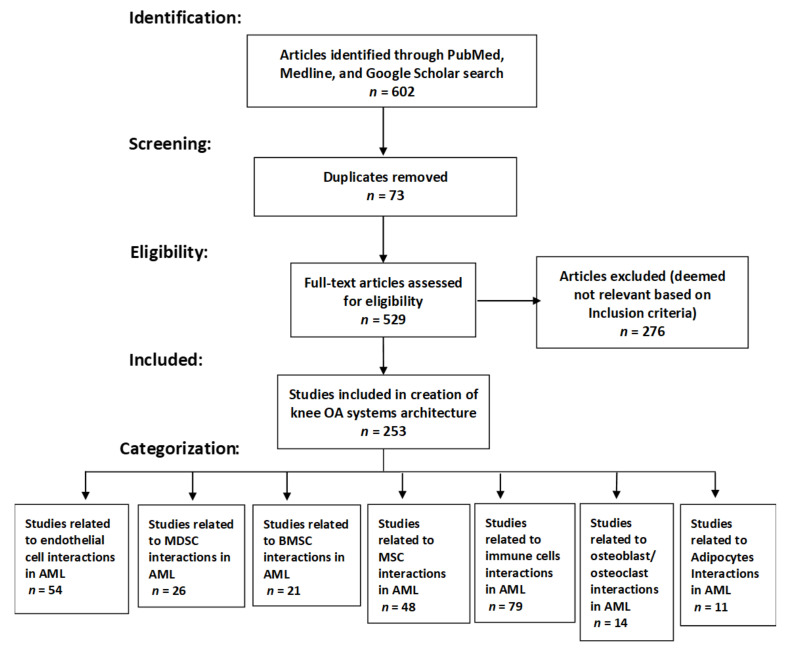
PRISMA flow diagram. In the process above, 602 articles are identified; 73 duplicates were removed; 529 articles were eligible for review from which 276 were removed as they were deemed not relevant; and 253 articles were included in the analysis.

The CytoSolve systematic bioinformatics literature review process and categorization are represented in Figure 1 as per the PRISMA guidelines [25]. We registered the systematic review with Research Registry. The unique identifying number assigned to our systematic review is: reviewregistry1290. Here is the link to the registry file: https://www.researchregistry.com/browse-the-registry#registryofsystematicreviewsmeta-analyses/registryofsystematicreviewsmeta-analysesdetails/61f5ba59a8ef6574c8e0f142/ (accessed on 18 April 2021).

## 3. Molecular Systems Architecture of AML

From the systems biology perspective, living organisms can be viewed as being comprised of dynamic networks of biochemical reactions [20]. The origin of disease is characterized by the disruption of one or more signaling cascades, which may arise due to defects at the molecular level and may ultimately result in the symptomatic manifestation of disease, due to gain or loss in the usual functions of the cascades involved [26]. The integration of molecular pathways acts as a backbone for the development of a molecular systems architecture for a disease [21]. In complex diseases, there are numerous cells involving different signaling cascades. In such cases, an integration of molecular pathway systems affecting these cell types results in a systems view of the disease or biological process.

**Figure 2:**
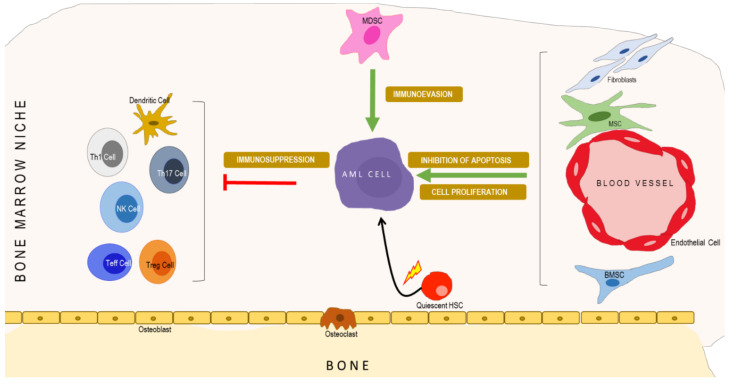
Stromal microenvironment in AML. The AML cell interacts with cells from the vascular niche, promoting cell proliferation and inhibiting the apoptosis of AML cells. MDSCs assist AML cells in evading the antitumor response from immune cells. AML cells, along with Tregs, suppress the immune response from the T cells and NK cells.

In Figure 2, we schematically illustrate a multilayered architecture of the AML microenvironment, with (i) an interconnected system of pathways in immune cells, endothelial cells, bone marrow stromal cells (BMSC), and myeloid-derived suppressor cells (MDSCs); (ii) converging points of key signaling pathways in the microenvironment, among AML cells, endothelial cells, BMSCs, MDSCs, and immune cells (interactive signaling layer); and (iii) the potential impact of such convergent pathways on the progression of AML (disease layer).

In AML, mutated leukemia stem cells (LSC) exploit the normal microenvironment and alter it to maintain their survival [27]. Alterations in the AML microenvironment can lead to AML relapse due to anti-apoptotic, anti-differentiation, and proliferative effects [28]. Stromal cells have a primary role in initiating AML, resulting in AML cells altering the normal localization and differentiation of HSCs as well as rapid leukemia growth expanding the intrinsically hypoxic microenvironment [29]. The microenvironment in AML consists of immune cells, stromal cells, and stem cells. Growth factors and cytokines released in the bone marrow (BM), thymus, and other immune tissue microenvironments provide paracrine and autocrine signals for long-term hematopoietic regulation of stem cells [30] and protect the AML cells from chemotherapeutic agents to promote drug resistance [31,32,33]. AML cells evade the immune cells by arresting the cell cycle of cytotoxic T cells, inducing cytotoxicity in NK cells and Th1 cells via tryptophan starvation [34,35].

## 4. Interactive Signaling in the AML Microenvironment

**Figure 3:**
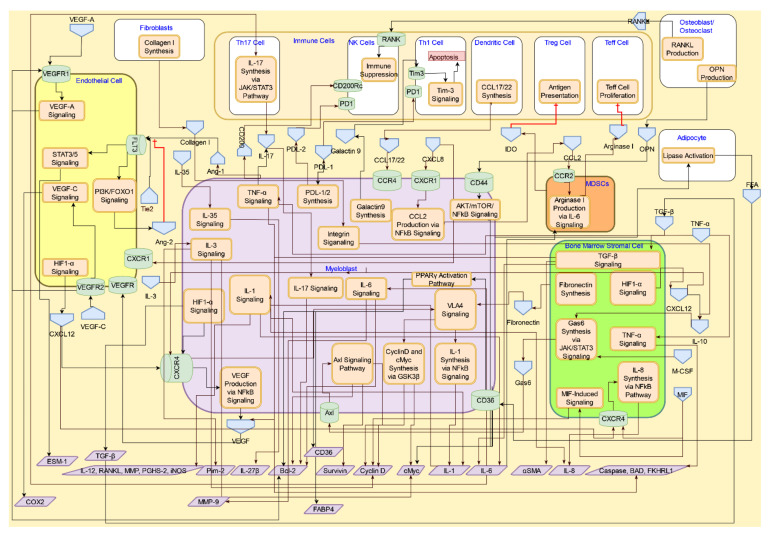
Schematics of interactive signaling between AML myeloblast and the cells of stromal microenvironment, such as bone marrow stromal cells (BMSC), myeloid-derived suppressor cells (MDSCs), immune cells, and endothelial cells derived from the CytoSolve bioinformatics process. A detailed exposition of the critical interactive signaling mechanisms is provided below. This exposition provides the critical elements of the AML molecular systems architecture.

The AML cells interact with the stromal cells to effect immunosuppression, immunoevasion, and survival/proliferation through promotion of inflammatory phenotypes in T cells, suppression of anti-inflammatory T cell phenotypes, and enhanced angiogenesis via a myriad of signaling transduction mechanisms. The signaling molecules that affect these processes can originate either from the leukemic cell or from the proinflammatory immune cells and other stromal cells; hence, they are important in developing a molecular systems architecture. CytoSolve’s bioinformatics process yields the schematic of the interactive signaling in the tumor microenvironment, as shown in Figure 3. Table 3 provides the legend describing the various graphical components of the systems architecture.

### 4.1. Interactive Crosstalk between AML Cells, Bone Marrow Stromal Cells, Endothelial Cells, Osteoblasts, and Adipocytes

#### 4.1.1. CXCR4/CXCL12 Signaling

The CXCR4/CXCL12 axis regulates retention of HSC quiescence, survival, and the size of the HSC pool in the marrow. It is also implicated in cellular migration, mobilization, and homing of LSCs during the initiation and progression of AML [36]. CXCR4 is a G protein-coupled chemokine receptor expressed on the surface of HSC and AML cells [29]. CXCR4 is essential for metastatic spread to organs and thereby allows tumor cells to access cellular niches, such as the bone marrow, that favor tumor-cell survival and growth. CXCL12 produced by the BMSCs, endothelial cells, osteoblasts, osteoclasts, and MSCs is a homeostatic chemokine that signals through CXCR4 and plays an important role in hematopoiesis and the development and organization of the immune system [37].

High levels of CXCL12 in hypoxic condition in the bone marrow niche indicate a regulator for the transcription factor, hypoxia inducibleg factor-1 (HIF-1) [38]. HIF-1α, in particular, is responsible for creating a concentration gradient of CXCL12 that guides malignant cell to the bone marrow niche and has been shown to upregulate the expression of CXCR4 on malignant cells [39]. Under normoxic conditions, HIF-1α is hydrolyzed by the prolyl hydroxylase domain protein (PHD), which leads to its ubiquitination [40]. The function of PHD is catalyzed by IDH and mutations in IHD have been shown to increase the accumulation of HIF-1α [40,41]. These results indicate that mutation in IHD may cause diminishing activity of IHD, leading to downregulation of PHD activity and higher levels of HIF-α. Another mutated gene in AML, FLT3-ITD, also has been shown to upregulate the translation of HIF-1α [41]. Mutation in FLT3-ITD leads to activation of FLT3 signaling [40], which upregulates the PI3K/AKT/mTOR pathway responsible for translation of HIF-1α [41,42]. These observations indicate a link between AML oncogenes IDH and FLT3-IDT and upregulation of the HIF-1α-induced CXCR4/CXCL12 axis signaling.

Secretion of functional CXCL12 from human BMSCs is a contact-dependent event mediated by connexin-43 and connexin-45 gap junctions [43]. The binding of CXCL12 to CXCR4 leads to activation of the PI3K/Akt and MAPK pathways that mediate the survival and proliferation of AML cells. CXCL12 also activates the NF-κB pathway, which induces the production of soluble factors, such as matrix metalloproteinases (MMPs), IL-8, and VEGF, leading to the angiogenesis promoted by MMPs and VEGF, and drug resistance initiated by IL-8 [32]. These soluble factors help degrade the extracellular matrix and induce blood vessel formation [37].

CXCL12 derived from MSCs has been shown to induce production of autophagy proteins such as ATG1, ATG5, and LC3 in the AML cells, which allows the AML cell to survive under stress [34]. MSCs-derived CXCL12 also upregulated the expression of the drug resistance protein P-glycoprotein (P-gp) via the PI3K/Akt/p38-MAPK pathway in the AML cells [44]. The ubiquitous nature of the CXCL12/CXCR4 axis in the AML microenvironment makes it a prime target for anticancer therapies [45]. Several therapeutics are under development, including those that inhibit or downregulate the expression of CXCR4 [46], inhibit the binding of CXCL12 to CXCR4 [47], and prevent the binding of CXCL12 to CXCR4 [48].

In the bone marrow niche, osteoblasts and osteoclasts lining the endosteum regulate bone formation and resorption [49]. During leukemogenesis, AML cells migrate to the bone marrow niche, due to the CXCL12 gradient created by osteoblasts and osteoclasts, [50] and evade detection [49].

**Figure 4:**
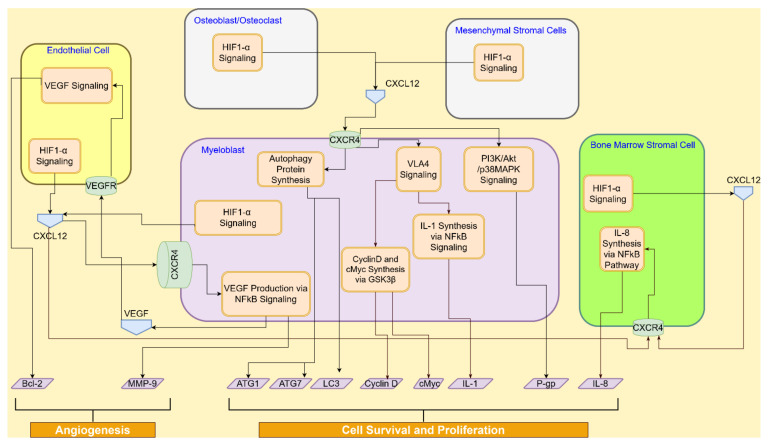
CXCR4/CXCL12 signaling interactions between myeloblast, endothelial cell, MSC, and BMSC promote angiogenesis in vascular niche, AML cell survival, and tumor proliferation.

The CXCR4/CXCL12 signaling pathway’s interactions across the AML cell, endothelial cell, osteoblasts/osteoclast, MSC, and the BMSC are shown in Figure 4.

#### 4.1.2. TGF-β Signaling

**Figure 5:**
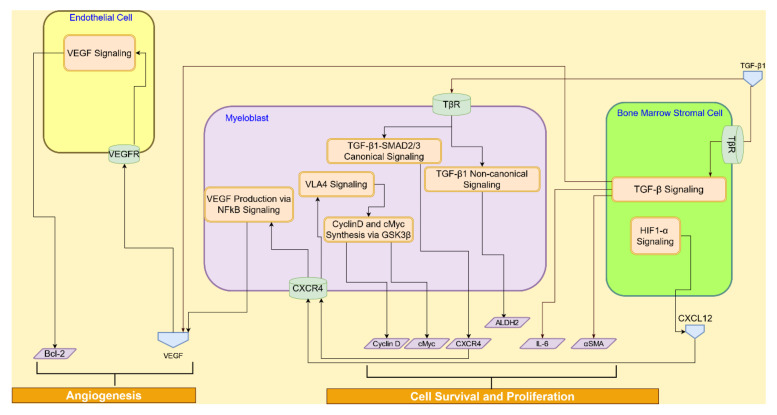
TGF-β signaling interactions between myeloblast, endothelial cell, and BMSC promote angiogenesis, AML cell survival, and tumor proliferation.

The multifunctional TGF-β regulates cell proliferation, survival, and apoptosis [51]. The three major mammalian TGF-β isoforms are TGF-β1, TGF-β2, and TGF-β3. TGF-β1 is the most abundant, universally expressed isoform. Once activated, the TGF-β ligands regulate cellular processes by binding to two ubiquitously expressed, high-affinity cell-surface receptors—type I receptor (TβRI) and type II receptor (TβRII)—both of which contain a serine/threonine protein kinase in their intracellular domains. Once bound to TGF-β, TβRII recruits, binds, and phosphorylates TβRI, thereby stimulating its protein kinase activity [52]. The activated TβRI then recruits and phosphorylates the receptor-activated transcription factors, Smad2/3, which then bind to the common Smad4, translocate into the nucleus, and interact in a cell-specific manner with transcription factors, coactivators, and corepressors to regulate the transcription of TGF-β-responsive genes [53]. The TGF-β signaling interactions across the AML cell, endothelial cell, and the BMSC, are shown in Figure 5.

TGF-β1 stimulates the secretion of IL-6 by BMSC and VEGF by AML cells, which in turn promotes the survival of AML cells and angiogenesis, respectively [54]. The TGF-β–Smad pathway is also known to induce the production of the extracellular matrix component fibronectin and the expression of integrin receptors in tumor cells, which facilitate cell adhesion and the cell-to-cell interaction of tumor cells with the extracellular matrix of BMSCs [51]. TGF-β1 induces expression of the chemokine receptor CXCR4 through activation of Smad2/3 [55]. CXCR4 is highly expressed in AML, and the interactions between CXCR4 and its ligand CXCL12, constitutively secreted by BMSCs and MSCs, promote the proliferation, survival, migration, and homing of cancer cells [36]. TGF-β1-triggered nuclear translocation of Smad2/3 regulates IL-6 and αSMA transcription, whereas HIF-1α translocation regulates VEGF and TGF-β1 transcription [56,57]. BMSC-derived TGF-β1 also induces the expression of aldehyde dehydrogenase-2 (ALDH2) via the non-canonical TGF-β-p38-ALDH2 pathway [58]. ALDH2 is implicated in conferring AML cells with drug resistance to chemotherapy [58].

The role of AML cells and the pro-angiogenic factor VEGF secreted by AML cells in promoting angiogenesis is dependent on the microenvironmental niche (e.g., the bone marrow niche, the vascular niche, etc.) and the progression of the disease. In the bone marrow niche, even though angiogenesis occurred, the resulting blood vessels were shown to be abnormal, leading to toxic levels of nitric oxide (NO)/reaction oxygen species (ROS) production and resulting in vascular regression [59]. In addition, as the disease progresses, the failed vasculature allows the AML cells to maintain low oxygen levels and to evade the chemotherapeutics, both of which are carried through blood [59]. Thus, unlike the solid tumors, the presence of VEGF in AML may not lead to angiogenesis of a functional vasculature.

#### 4.1.3. RANK/RANKL and Osteopontin Signaling in Osteoblasts/Osteoclasts

The RANK/RANKL pathway governs bone remodeling. The receptor RANK is on the surface of osteoclasts, and its ligand RANKL is expressed on the membranes of osteoblasts, and also secreted by activated lymphocytes and AML cells [60]. The binding of RANKL to RANK initiates osteoclastogenesis and increases the survival of osteoclasts [61]. RANK is also expressed on natural killer (NK) cells. RANKL expressed in AML cells binds with RANK on NK cells to compromise their anti-leukemic activity [60].

**Figure 6:**
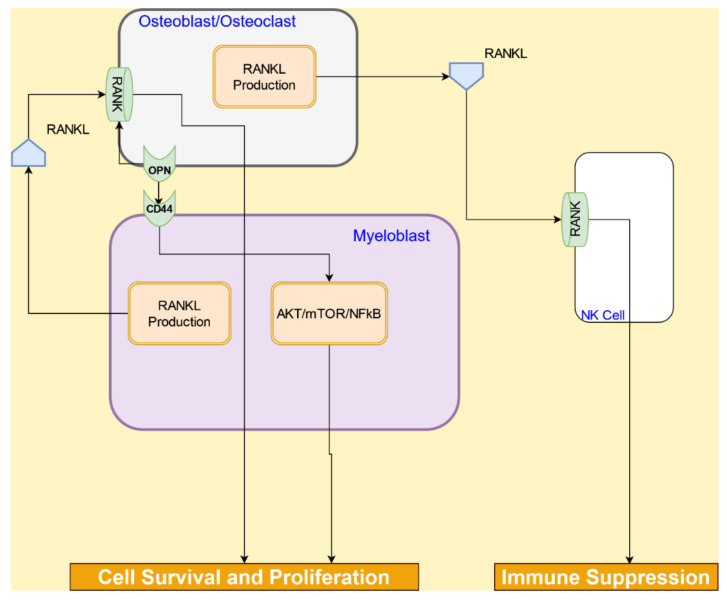
Interactions between osteoblasts/osteoclasts and AML cells lead to AML cell survival, proliferation as well as immune suppression.

Osteopontin (OPN), an extracellular matrix protein expressed on osteoblasts and osteoclasts, was found to be increased in the serum of patients’ AML [62]. OPN promotes the survival and proliferation of AML blasts through its binding to CD44 on the AML cell surface, which subsequently initiates the AKT/mTOR/NF-*κ*B signaling pathways [62,63]. The RANK/RANKL and OPN/CD44 pathways are shown in Figure 6.

Adipose tissue, which accounts for up to 70% of the bone marrow, acts as a reservoir for HSCs and progenitor cells [64]. Bone marrow adipocytes have been shown to support the survival and proliferation of AML cells in vivo and in vitro [65]. AML blasts induce the activation of lipolysis in the adipocytes by promoting phosphorylation of the lipase, leading to free fatty acid release [65]. Lipolysis is initiated by activation of the β-adrenergic receptor [65], leading to stimulation of hormone-sensitive lipase (HSL) in the presence of AML blasts [65,66].

**Figure 7:**
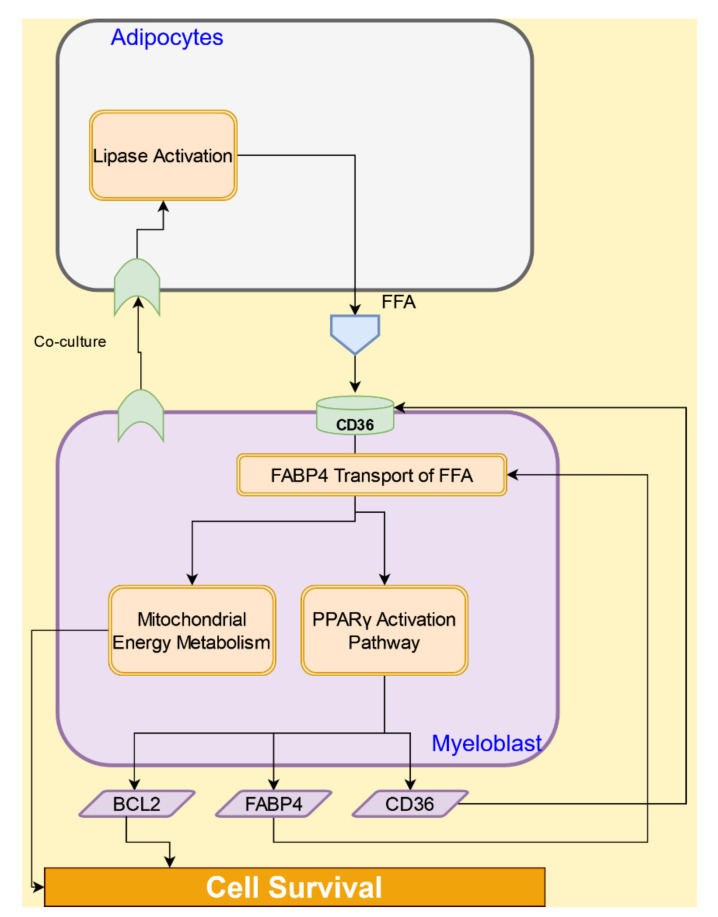
Interactions between adipocytes and AML cells lead to AML cell survival.

The free fatty acids released by adipocytes are internalized by the CD36 receptor on the AML cells and subsequently transferred to the nucleus and mitochondria by the intracellular lipid chaperone fatty acid binding protein 4 (FABP4). In the nucleus, the free fatty acid activates the transcription factor PPARγ, which induces the transcription of fat transport-associated genes, such as *CD36* and *FABP4*, and the anti-apoptotic gene BCL2 [67]. In the mitochondria, fatty acids are used as a source of energy via the metabolic activation of fatty acid oxidation (FAO) and oxidative phosphorylation [67]. The interactions between adipocytes and AML cell are illustrated in Figure 7.

### 4.2. Interactive Crosstalk Signaling between AML Cells and Endothelial Cells via Adhesion Molecules

Interactions between adhesion molecules, such as VCAM-1 and E-selectin, on endothelial cells and their ligands, expressed on HSCs/AML cells and the marrow niche, mediate the retention of HSCs and AML cells within the bone marrow niche [68]. Very late antigen-4 (VLA-4), also known as integrin α4β1, is a heterodimer expressed on leukocytes and variably on AML blasts [69]. In addition, In addition, VCAM-1, which is a ligand for VLA-4 on AML cells, is also expressed by osteoblasts and endothelial cells [70]. Under normal circumstances, CXCL12 stimulation results in the activation of VLA-4 on HSCs, leading to activation of the VLA-4/VCAM-1 signaling pathway, and enhancement of HSC adhesion to the endothelial cells followed by their trans-endothelial migration [71]. Interactions of AML cells with endothelial cells and their subsequent integration and proliferation in the vascular niche are facilitated by the VLA-4/VCAM-1 axis [29].

**Figure 8:**
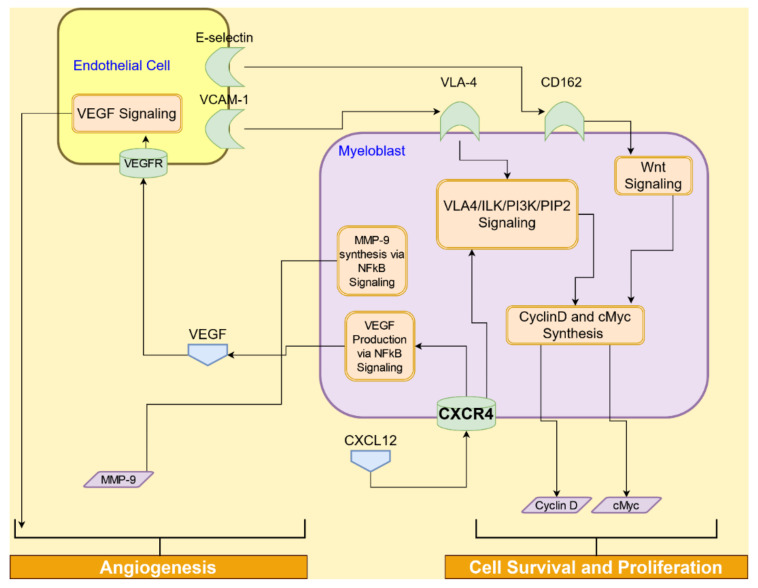
Signaling between endothelial cells and AML cells (myeloblast) promotes adhesion of AML cells in the vascular niche, survival, proliferation, and attempted angiogenesis.

The VCAM1-VLA-4 signaling, as illustrated in Figure 8, promotes cell survival and proliferation by interfering with the activation of receptor tyrosine kinase (RTK) [72]. A main structural and signaling protein, integrin-linked kinase (ILK), binds with VLA-4. ILK forms multiprotein complexes with several key components involved in intracellular signaling cascades [73]. ILK kinase activity is dependent on PI3K and requires binding of phosphatidylinositol (3,4,5)-trisphosphate (PIP3). Next, glycogen synthase kinase-3β (GSK3β) is phosphorylated by ILK at serine 9 residue, leading to the activation of the activator protein 1 (AP-1), which then upregulates cyclin D1 and Myc-1 [74]. Thus, the VCAM1-VLA-4 signaling pathway plays a critical role in the survival and proliferation of leukemic cells.

CXCL12/CXCR4 signaling in AML cells activates the NF-κB pathway, which induces the production of MMPs and VEGF, leading to angiogenesis [32]. These soluble factors help degrade the extracellular matrix and induce blood vessel formation [37]. In the endothelial cells, VEGF signaling leads to glycolysis-mediated vascular remodeling [58]. Targeting VEGF receptor-2 with a tyrosine kinase inhibitor resulted in AML cytotoxicity, due to inhibition of VEGF-induced survival signaling and vascular remodeling in the tumor microenvironment [34].

E-selectin, a cell adhesion molecule, regulates the rolling of leukocytes over endothelial cells [75]. Its ligand, E-selectin ligand-1 (ESL-1), is found on HSCs as well as AML blasts [76]. E-selectin directly regulates disease progression and chemoresistance in AML [77]. AML blasts’ survival is enhanced by their adhesion to the vascular niche via ESL-1, which activates Wnt signaling [78]. Inhibition of E-selectin binding to AML blasts augmented chemotherapeutic effect and lowered the vascular niche-mediated survival of AML blasts [76]. Recently, CD162 has emerged as another E-selectin ligand that is implicated in the chemoresistance of AML [77].

### 4.3. Interactive Signaling with Myeloid-derived Suppressor Cells (MDSCs) in AML

**Figure 9:**
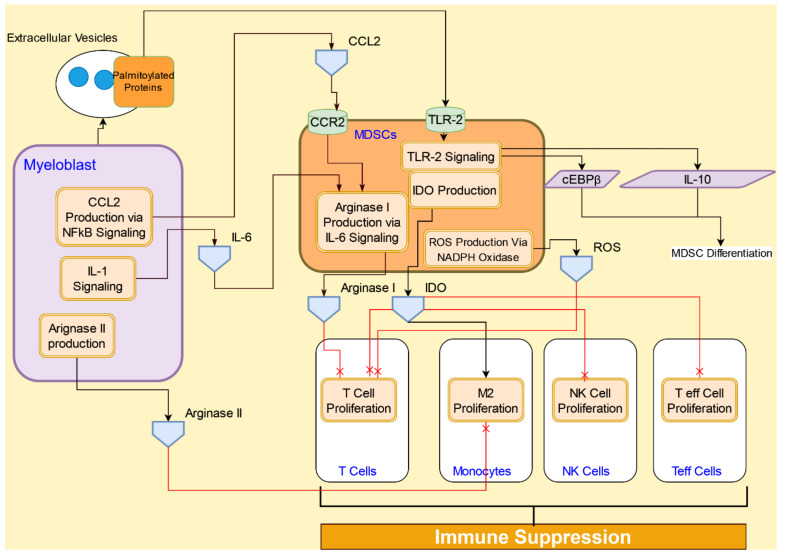
AML cells’ interactions with MDSC cells lead to suppression of immune cell proliferation.

MDSCs are critical to the immunosuppressive characteristic of the tumor microenvironment and are involved in promoting immune tolerance and disease growth. AML patients show significant increase in MDSCs in the circulation, and leukemic blasts directly induce the expansion of MDSCs [79]. MDSCs are the nonmalignant immature myeloid cells, whereas AML blasts are a malignant expansion of immature myeloid cells; both have the ability to suppress immune cells [80]. A key factor that characterizes the interactions between an AML cell and MDSC is the oncogene MUC1. The expression of MUC1 on the leukemic blasts and leukemia-initiating cells induces MDSC expansion in the microenvironment. The silencing of MUC1 has been shown to reduce the capacity of AML blasts to induce MDSC expansion in the tumor microenvironment [79]. Interactions of MDSC with the AML blasts and immune cells are illustrated in Figure 9.

Immunosuppression by MDSCs and AML blasts is regulated through several mechanisms. One of the key mechanisms includes immunosuppression by the enzyme arginase present in both MDSCs and AML blasts [81]. MDSCs express arginase I and AML blasts express arginase II. The two isoforms of arginase are likely to have resulted from a gene duplication event during evolution, with arginase I located in the cytosol and arginase II in the mitochondria [82]. Both arginase isoforms convert arginine into urea. The two enzymes catalyze the same reaction, converting arginine into ornithine, with urea as a byproduct. In healthy individuals, arginase I is expressed predominantly by hepatocytes, whereas arginase II is expressed in a more diverse range of organs [82].

AML blasts express and release arginase II to suppress T-cell proliferation via depletion of L-arginine in the microenvironment [83]. The combination of increased intracellular arginase activity and plasma arginase activity halts T-cell proliferation and contributes to the lymphopenia. In addition, arginase II levels and activity serve as important biomarkers in patients with AML. The measurement of arginase II levels acts as a biomarker for minimal residual disease. AML blasts polarize neighboring monocytes to an immunosuppressive M2-like phenotype [81].

MDSCs also synthesize indoleamine-pyrrole 2,3-dioxygenase (IDO), a tryptophan-degrading enzyme, and contribute to immune tolerance by mediating T cell suppression. IDO locally depletes tryptophan and generates tryptophan metabolites, including kynurenine, resulting in reduced proliferation of CD4^+^ T cells, CD8^+^ T cells, and natural killer (NK) cells [84]. ROS secretion also contributes to the immunosuppressive action of MDSC and is caused by the increase in NADPH oxidase activity in granulocytic MDSC [85]. A subset of MDSCs deplete cysteine as an alternate mechanism of immune suppression. MDSCs also modulate the surrounding macrophages and dendritic cells [86].

The post-translational addition of a palmitate to a protein creates greater affinity for non-polar structures, such as lipid bilayers, and are critical to the functioning of normal as well as cancer cells [87]. In AML cell-derived extracellular vesicles, carrying palmitoylated proteins leads to monocytes differentiating into MDSCs. This process is regulated by TLR-2 signaling, leading to upregulation of cEBPβ and IL-10 expression [34].

### 4.4. Interactive Signaling AML Cells and Immune Cells

In the AML microenvironment, the AML cells directly interact with several immune cells, including Treg cells, NK cells, Th-1 cells, dendritic cells (DC), and T effector (Teff) cells [88]. The interactions between AML cells and the immune cells are described below.

#### 4.4.1. Interferon α Signaling

Interferon-α (IFN-α), a type I IFN, has been previously used as an antitumor agent [89]. Type I IFNs exert direct antitumor effects on AML cells by multiple mechanisms modulated via the expression of interferon-inducible genes. IFN-α inhibits the production of pro-proliferative cytokines such, as IL-1 and IL-6, and pro-angiogenic cytokine IL-8 [89]. IFN-α also promotes the expression of FasL in AML cells, which initiates apoptotic signaling via caspase-8 [90]. IFN-α is also implicated in the activation of DCs, NK cells, and T cells, which in turn play major roles in antitumor immune responses [91].

**Figure 10:**
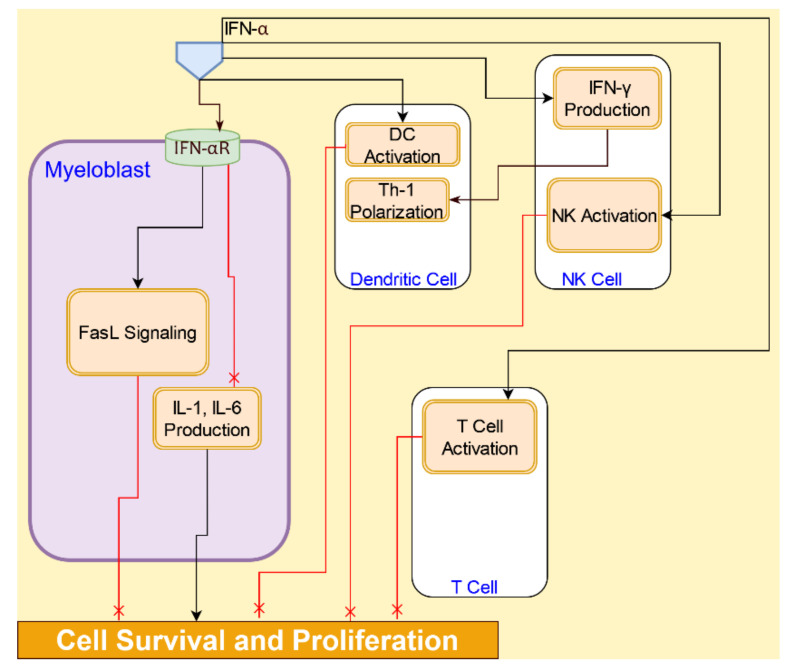
IFN-α signaling in the tumor microenvironment leads to activation of immune response and inhibition of AML cell survival and proliferation.

Mutation in cohesin complex proteins, seen in AML cells, downregulates Type I IFNs in macrophages [92]. Type I IFNs are critical to the initiation of antitumor immunity through direct actions on DCs. IFNα induces DCs to exert direct cytotoxic activity against AML cells [93]. IFN-α has an important role in modulating NK cell function [91]. IFN-α upregulates the expression of immunomodulatory cytokines, such as IFN-γ in the NK cells, promoting their “helper” function. The helper NK cells induce the DCs with Th-1-polarizing capacity, which is necessary for antitumor immunity [89]. Activation of the immune system by interferons has been shown to undermine AML cell growth [11,89,93] The IFN-α signaling interactions are illustrated in Figure 10.

#### 4.4.2. Immunosuppressive Interactions of Tregs in AML Tumor Microenvironment

Tregs play a pivotal role in maintaining peripheral immunological tolerance by preventing autoimmunity and chronic inflammation. There are two subtypes of Tregs: naturally occurring Tregs (nTregs) and induced Tregs (iTregs) [94]. iTregs start out as CD4^+^ cells and acquire CD25 and FoxP3 expression following adequate antigenic stimulation in a specific tolerogenic microenvironment [95]. AML patients with a higher expression of IDO critically induce a de novo population of Foxp3^+^ Tregs [96]. AML cells have also been shown to actively recruit and program Treg to suppress antitumor immune responses [97].

AML cells promote the expansion of Tregs via the inducible T-cell costimulator ligand (ICOSL). TNF-α signaling induces ICOSL expression in AML cells [98]. The accumulation of Tregs in the AML microenvironment is driven by the chemotactic effect of CCL2 [88]. Once established in the microenvironment, Tregs actively prevent or downregulate antitumor responses from the immune cells in the tumor microenvironment. Tregs suppress the immune response from Teffs through two mechanisms: a contact-dependent manner, and a contact-independent manner. While nTregs use both mechanisms, iTregs induce immunosuppression in a contact-independent manner that involves cytokines, such as IL-4, IL-10, or TGF-β [97,98]. In addition to suppressing APCs, IL-10 also promotes AML cell proliferation via the ERK/p38/STAT3 pathway [98]. Treatment with an FLT3 inhibitor, midostaurin, showed a significant decrease in the Treg population, reduction in the FOX3p mRNA expression in AML cells, and reduction in IL-10 levels, indicating a role for IL-10 as a potential biomarker for AML cancer treatment [99].

Direct cell-to-cell interactions between Tregs and Teffs result in apoptosis and/or suppression of Teffs. The direct transfer of cAMP from Tregs to Teff through the gap junctions leads to downregulation of IL-2 production and subsequent proliferations of Teff [100]. On contact, the formation of gap junctions occurs between Tregs and Teffs. cAMP transferred through the gap from Tregs to Teffs suppresses the proliferation of Teffs by decreasing IL-2 production.

**Figure 11:**
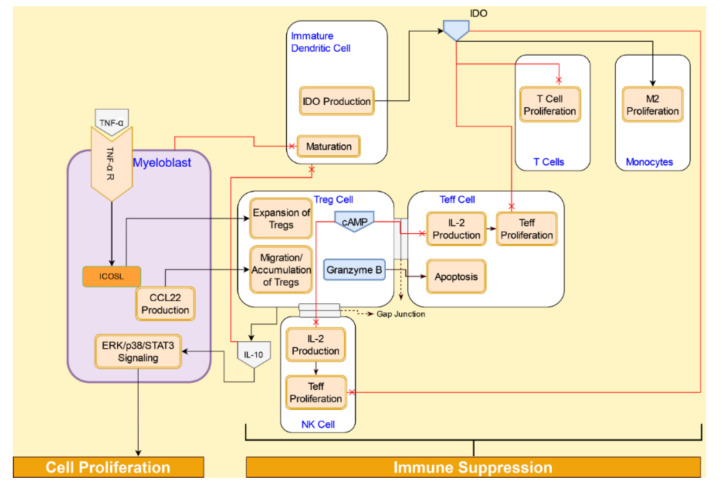
Tregs signaling in the tumor microenvironment promotes immuno-evasion by AML blasts via suppression of immune cell proliferation and promotion of immune cell apoptosis.

Apoptosis of CD4^+^CD25^+^ Teffs is caused by Tregs through the granzyme B-dependent and perforin-independent mechanisms [101]. Tregs also suppress NK-cell proliferation via depletion of IL-2 [101]. Tregs block the maturation of DCs. The immature DCs express IDO, which depletes tryptophan needed for T-cell proliferation [102]. Metabolites resulting from IDO depletion of tryptophan, such as kynurenines, actively promote T-cell apoptosis [97]. The immunosuppression by AML cells through Tregs is illustrated in Figure 11.

#### 4.4.3. Immunosuppression Interactions of AML Cells with T Cells

The programmed death-1 (PD-1) receptor is expressed on various cell types, including T cells [103]. In AML, an increased expression of PD-1 receptor is observed in cytotoxic T lymphocytes (CTL). PD-L1, a ligand for the PD-1 receptor, is present on cancer cells, including AML [104]. PD-1 and PD-L1 interaction suppresses CTL response to AML blasts [97]. PD-L1 expression was found to be higher in patients undergoing chemotherapy or those who have a relapse, suggesting a refractory role for PD-1/PD-L1 interaction [105].

**Figure 12:**
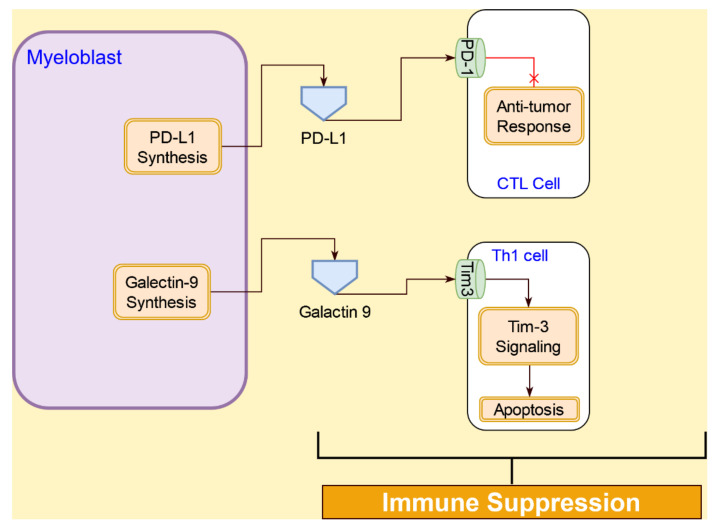
Immunosuppression of T cells by AML blast in the tumor microenvironment is mediated through PD-L1/PD-1 signaling and Tim-3/Galectin 9 signaling.

AML blasts also participate in the suppression of Th cells through Tim-3 and galactin-9 (gal-9) interactions [106]. A type I membrane glycoprotein, Tim-3 is expressed on Th1 cells and innate immune cells [107]. Gal-9 is expressed on AML cells and participates in the Tim-3/gal-9 pathway that leads to apoptosis of Th1 cells [108]. In addition, the Tim-3/gal-9 pathway, along with the PD-1/PD-1 ligand pathway, is involved in regulating CD8^+^ CTL responses [109]. The interactions between AML blasts and Th1, as well as CD8^+^ CTLs, are illustrated in Figure 12.

#### 4.4.4. Interactive Signaling with Natural Killer (NK) Cells in AML

NK cells are lymphocytes from the innate immune system. NK cells can directly eliminate tumor cells via their cytotoxic and cytokine-secreting capacity and indirectly contribute to tumor control by communicating with DC and other immune cells, supporting the development of an efficient adaptive antitumor immune response [110].

**Figure 13:**
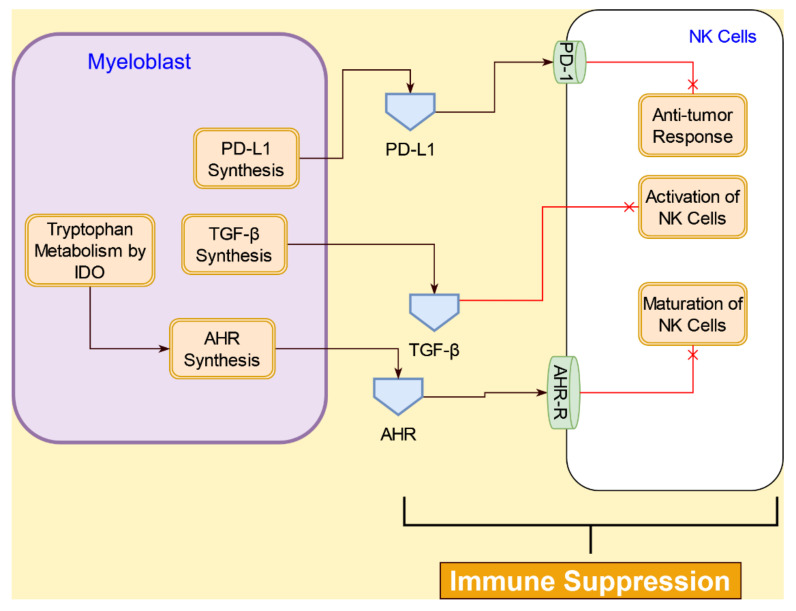
Interactions between AML cells (myeloblast) and NK cells in the tumor microenvironment lead to immune suppression of NK cells mediated by antitumor response through PD-L1/PD-1 signaling inhibition of NK cell activation via TGF-β signaling.

NK cells mediate their antitumor activity through the expression of several chemokine receptors, such as CCR1, CCR4, CCR6, CCR7, CXCR1, CXCR3, CXCR4, CXCR6, and CX3CR1 [111]. Many of the ligands for these NK cell receptors are constitutively released by AML cells, including the chemokines within the CCL2–4/CXCL1/8 cluster found in most AML patients, indicating an expected migration of NK cells towards the AML blasts [112]. However, AML cells are able to evade NK cell immune surveillance through a number of mechanisms. The interactions between AML cells and NK cells are illustrated in Figure 13.

A dysfunctional antitumor immune response by the NK cell could result in NK cell abnormalities, immuno-suppressive and immuno-evasive properties of AML target cells, and preferential interactions with other immune cells rather than AML blasts [113]. In AML, changes in the expression of both receptors and ligands are commonly found, which substantially impair NK cell-mediated killing. The majority of AML patients have a downregulated NK cell surface expression of the activating natural cytotoxicity receptors; thus, AML cells evade NK cells’ mediated killing by the lowered or absent expression of surface ligands (e.g., CD48, NKG2DL, etc.) for various NK cell activating receptors [114]. NK cells can also be inactivated by soluble inhibitory factors, such as TGF-β, and reactive oxygen species secreted by AML blasts [115,116]. However, patients with AML-ETO-positive AML have a better prognosis, as AML-ETO has been shown to upregulate the NK cell ligand CD48. This allows NK cells to perform cell-mediated killing of AML cells [117].

Another mechanism for AML cells to escape from NK cells is the activation of the aryl hydrocarbon receptor (AHR) pathway in the NK cells [118]. IDO, which is highly expressed in AML blasts compared to normal cells [91], initiates the AHR activation by kynurenine. The active AHR binds to the AHR receptor on naïve NK cells and leads to the expression of miR-29/b1, which blocks the NK cell differentiation [118], thereby allowing the AML cells to escape from the NK cells. Inhibition of AHR has been shown to restore the NK cell-mediated killing of AML cells [118], indicating a potential role for AHR as a therapeutic target.

## 5. Discussion

**Figure 14:**
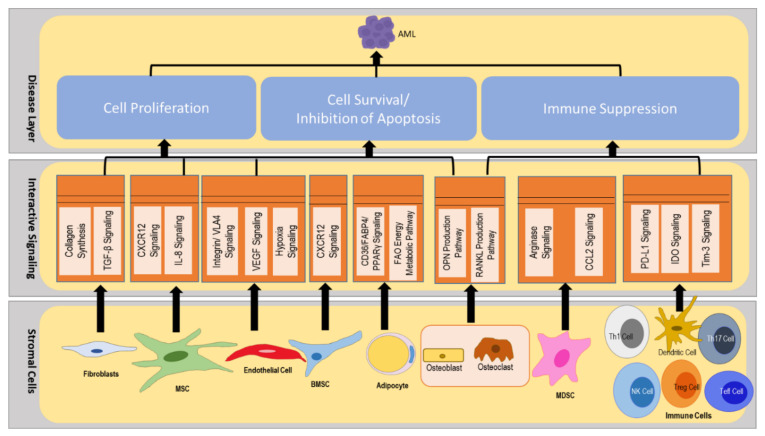
Molecular systems architecture of interactive signaling in the AML stromal microenvironment. In the three-layered architecture, the bottom layer consists of stromal cellular factors involved in the pathogenesis of AML. The middle layer consists of the stromal interactions within and among the cellular components. The top layer represents the biological processes resulting from the interactions in the stromal microenvironment. This molecular systems architecture provides a visual representation of the systems biology of AML based on the current science reviewed and curated. The architecture provides a framework for scientific collaboration and instantiation of future knowledge, based on new science and feedback from the AML community.

The CytoSolve systematic bioinformatics review identified critical molecular systems components of AML pathogenesis. The organization of these components into a molecular systems architecture is presented in Figure 14. The first layer, starting from the bottom of Figure 14, represents cellular components of the stromal microenvironment: fibroblast, MSCs, endothelial cells, BMSCs, MDSCs, and immune cells. The second layer, in the middle of Figure 14, represents the key molecular interactions implicated in the pathogenesis of AML: collagen synthesis and TGF-β signaling in fibroblasts; CXCL12 signaling and IL-8 signaling in MSCs; VLA4 signaling, VEGF signaling, and hypoxia signaling in endothelial cells; CXCL12 signaling in BMSCs; CD36/FABP4/PPARγ signaling and the FAO energy metabolic pathway in adipocytes; the OPN production pathway and the RANKL production pathway in osteoblast/osteoclast; arginase signaling and CCL2 signaling in MDSCs; and PD-L1 signaling, IDO signaling, Tim-3 signaling, and IL-17 signaling in immune cells. The third layer, shown at the top of Figure 14, represents the biological processes implicated in the pathogenesis of AML: angiogenesis; cell proliferation, cell survival (inhibition of apoptosis), and immune suppression.

The molecular systems architecture in Figure 14 provides a consolidated guide to understanding the overall AML pathogenesis. Interactions among the nine cell types in the bottom layer give rise to the sixteen molecular systems presented in the middle layer. Of these sixteen molecular systems components, eight of them (collagen synthesis, TGF-β signaling, CXCL12 signaling, IL-8 signaling, the OPN production pathway, VLA4 signaling, VEGF signaling, and hypoxia signaling) contribute to AML pathogenesis by promoting cell proliferation and cell survival/inhibition of apoptosis. The remaining eight molecular systems components (arginase signaling, CCL2 signaling, PD-L1 signaling, IDO signaling, Tim-3 signaling, TLR-2 signaling, the AHR pathway, and RANK/RANKL signaling) contribute to AML pathogenesis by promoting immune evasion and suppression. The integrated processes of cell proliferation, cell survival, and immune suppression, driven by the molecular subsystems and the respective cellular interactions, give rise to AML.

The architecture also may offer a vehicle for new insights and discovery. For example, we have identified several targets across different cell types in the microenvironment that can potentially be used to develop therapeutic interventions to inhibit suppression of immune response, inhibit cell proliferation, and promote cancer cell apoptosis, as listed in Table 4.

Efforts are already underway to develop antibody conjugates for cell surface markers, such as CD44, CLL-1, CD34, and Tim-3 [119,120], and insights from this architecture can advance such efforts by the identification of new targets and understanding the mechanisms of action of new single and combination therapies based on their interactions with the targets.

## 6. Future Directions

Mechanistic in silico modeling is emerging as a valuable pre-clinical drug discovery tool. Molecular systems architecture, as presented in this study, provides a starting point for such mechanistic in silico modeling efforts. The computational capabilities of CytoSolve can be employed to create an integrative computational model for the AML stromal microenvironment. The resulting in silico AML stromal microenvironment model can then be used as a testing and validation platform to identify new targets and novel combination therapies.

## 7. Conclusions

The molecular systems architecture developed in this review provides a blueprint for understanding the complex interactions occurring in the AML microenvironment. This understanding will enable the identification of targets in the interactive signaling pathways that may be used to develop novel combination therapies and synthetic approaches that may be more effective than the current therapeutic options and may potentially mitigate undesirable side effects. The molecular systems architecture provides a versatile tool in identifying how targeting a particular mechanism in a stromal cell can a have either a positive or negative cascading effect on the rest of the stromal microenvironment, thereby providing a much better drug development paradigm that can minimize side effects and maximize efficacy of treatment. This architecture may also be converted to an open science interactive web-based tool to enable ongoing collaborative development by the AML research community. Such efforts have been done before in the field of human knee osteoarthritis [121].

## Figures and Tables

**Table 1:** Gene mutations in AML. Class I, Class II, and Class III genes are involved in signal transduction, differentiation, and epigenetic regulation, respectively. In addition, tumor suppression genes and other oncogenes are also implicated in AML pathogenesis.

Class I Genes	Class II Genes	Class III Genes	Other Genes	
Signal Transduction	Differentiation	Epigenetic Regulation	Tumor Suppression	Oncogenes
FLT3	RUNX1 (AML1)	TET2	WT1	PML-RARa
KIT	CBFα	IDH1/IDH2	TP53	FLT3-ITD
NRAS, KRAS	CEBPα	DNMT3A		AML-ETO
JAK2	NPM1	ASXL1		CBFB-MYH11
PTPN11	PU1	EZH2		
	MLL	Cohesin		
	RARα	NPM1		

**Table 2:** MeSH keywords used for computer-based screening.

MeSH Keywords
Human acute myeloid leukemia CXCR4 CXCL12 Signaling NOT review
Human acute myeloid leukemia TGF-β Signaling NOT review
Human acute myeloid leukemia VLA-4 VCAM-1 Signaling NOT review
Human acute myeloid leukemia Arginase NOT review
Human acute myeloid leukemia IDO NOT review
Human acute myeloid leukemia PD-1 PD-L1 Signaling NOT review
Human acute myeloid leukemia NK cells NOT review
Human acute myeloid leukemia BMSC NOT review
Human acute myeloid leukemia MDSC NOT review
Human acute myeloid leukemia Endothelial Cell NOT review
Human acute myeloid leukemia Treg cells NOT review
Human acute myeloid leukemia MSC cells NOT review
Human acute myeloid leukemia Fibroblast cells NOT review
Human acute myeloid leukemia Th1 cells NOT review
Human acute myeloid leukemia Th17 cells NOT review
Human acute myeloid leukemia Teff cells NOT review
Human acute myeloid leukemia Osteoblasts/Osteoclast cells NOT review
Human acute myeloid leukemia Adipocytes NOT review

**Table 3:** Legend of symbols used in Figure 3, Figure 4, Figure 5, Figure 6, Figure 7, Figure 8, Figure 9, Figure 10 and Figure 11.

Name of Symbol	Symbol	Description
Double-sided Orange Rectangle	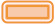	Molecular pathway
Black Arrow	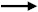	Receptor/Ligand Binding, Signal propagation
Red Flat Arrow	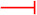	Inhibition of signal propagation
Green Cylinder	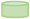	Cell surface receptor
Purple Lozenge	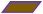	mRNA
Blue Pentagram	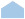	Protein/small molecule

**Table 4:** Summary of potential therapeutic molecular targets. The targets are categorized according to physiological effects. Ten molecular targets were identified in the molecular mechanisms involved in suppression of immune response across AML cells, Th1 cells, NK cells, and MDSC cells. Three targets were identified in BMSC and two in osteoblast/osteoclast in the molecular mechanisms involved in cell proliferation. Three targets in AML cells and one target in adipocyte were identified in the molecular mechanisms involved in cell apoptosis.

Physiological Effect	Cell Type	Potential Target
Suppression of Immune Response	AML Cell	PD-L1, IL-6, Galactin-9, CCL2, CXCR1, IDO
	Th1-Cell	Tim-3, PD-1
	NK Cell	PD-1, AHR
	MDSC	Arginase, CCR2
Cell Proliferation	BMSC	Fibronectin, Gas-6, CXCR4/CXCL12
	Osteoblast/Osteoclast	OPN, CXCR4/CXCL12
Cell Apoptosis	AML Cell	Axl, IL-17, IL-6
	Adipocytes	FAO
